# A fast‐growing schwannoma of the tongue in a 15‐year‐old Iranian male: Review of literature and case report

**DOI:** 10.1002/ccr3.4266

**Published:** 2021-06-24

**Authors:** Farzaneh Agha‐Hosseini, Mahdieh‐Sadat Moosavi, Pouyan Aminishakib, Marzieh Yousefian

**Affiliations:** ^1^ Department of Oral and Maxillofacial Medicine Dental Research Center School of Dentistry Tehran University of Medical Sciences The Academy of Medical Sciences Tehran Iran; ^2^ Department of Oral and Maxillofacial Medicine Dental Research Center School of Dentistry Tehran University of Medical Sciences Tehran Iran; ^3^ Department of Oral and Maxillofacial Pathology School of Dentistry Tehran University of Medical Sciences Tehran Iran; ^4^ Department of Oral and Maxillofacial Medicine School of Dentistry Alborz University of Medical Sciences Karaj Iran

**Keywords:** neurilemoma, oral cavity, schwannoma

## Abstract

Schwannoma can be included in the list of differential diagnoses of tongue masses but seems to be a rare finding in the Iranian population. The current case was presented as an exophytic nodular sessile mass which was growing fast.

## Graphical abstract

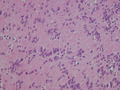

## 1 INTRODUCTION

Schwannomas are encapsulated benign tumors with a slow growth rate, which are often single. Intraoral schwannoma is a rare finding. Herein, we report the first case of schwannoma of the tongue in a 15‐year‐old Iranian male patient.

Schwannoma, also referred to as neurilemmoma, is a benign, encapsulated tumor with a slow growth pattern, which is often single and originates from the Schwann cells wrapping around the axons of neurons in peripheral, cranial (except for the optic and olfactory nerves), spinal, and autonomic nerves. Around 25%‐40% of schwannomas occur in the head and neck region; of which, only 1%‐12% occur intraorally.^1^ Schwannomas can occur independently or as part of a genetic disorder such as neurofibromatosis 1 and 2, and schwannomatosis.^2^ The Schwann cells originate from the neural crest cells. All axons of the peripheral nervous system are wrapped with Schwann cells. The Schwann cells have two different types:

1. The myelinating Schwann cells that produce the myelin sheath wrapping around the peripheral axons.

2. The nonmyelinating Schwann cells that play a fundamental role in response to injury to the peripheral nervous system. They produce neurotropic factors and lead to regeneration of the injured neurons. They also guide the immune response in the peripheral nervous system.^3^ Several case reports are available in the literature regarding oral schwannomas. However, search of the literature by the authors did not yield a comprehensive review on this topic. Since 1954, only six confirmed cases of schwannomas in the Iranian patients have been reported in the literature. This case report describes a case of schwannoma of the tongue in a 15‐year‐old Iranian male and reviews the related literature regarding oral schwannomas published in the past 66 years.

The current case is reported in line with the SCARE criteria.^4^ An electronic search of the literature was carried out in the PubMed and Google Scholar databases using the keywords “oral neurilemmoma”, and “oral schwannoma”. The search was limited to case series, case reports, and literature reviews in English, published from 1954 to 2020. The following information was extracted from the retrieved articles: chief complaint of patients, age and gender of patients, location of lesions, and objective of the reports.

The patients were then classified into three groups of children, adolescents, and teenagers (≤20 years of age), middle‐aged individuals (>20 and ≤60 years), and the elderly (>60 years) based on their age range.

## 2 CASE REPORT

**FIGURE 1:**
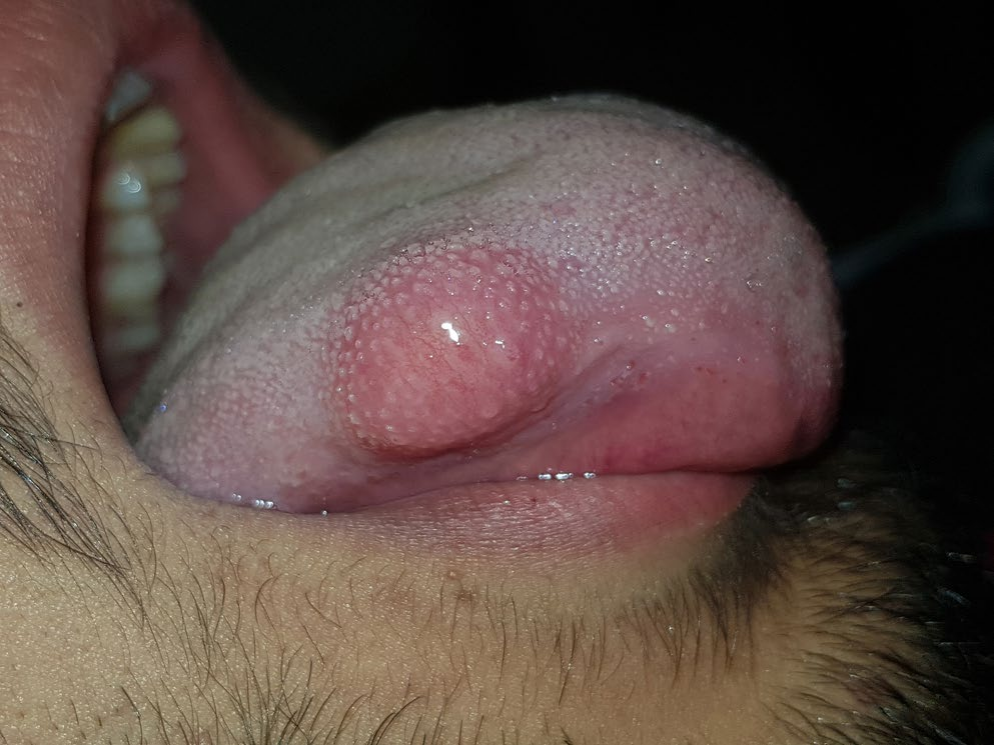
Preoperative view of an exophytic nodular mass

Our patient was a 15‐year‐old Iranian male who presented to the Oral and Maxillofacial Medicine Department of School of Dentistry, complaining of a painless swelling on his tongue from 1 month earlier, which was growing fast (Figure 1). He did not recall any history of trauma or tongue biting. His medical history was unremarkable, and he was not using any medication.

He did not have any symptoms such as pain, paresthesia, altered sense of taste, or mouth dryness. Intraoral examination revealed an exophytic nodular sessile mass with an intact surface with the same color as the surrounding mucosa, measuring 1.5 × 1.5 cm in size. It had a firm consistency and was located in the posterolateral surface of the tongue, extending to the ventral surface. The lesion was freely moving beneath the covering mucosa. The patient had no lymphadenopathy.

Based on the patient's history and clinical findings, a magnetic resonance image (MRI) of the tongue without contrast was requested for the patient, which revealed a tongue mass with well‐defined borders measuring 15 × 12 mm, which was hypersignal on T2‐weighted, and hyposignal on T1‐weighted images.

**FIGURE 2:**
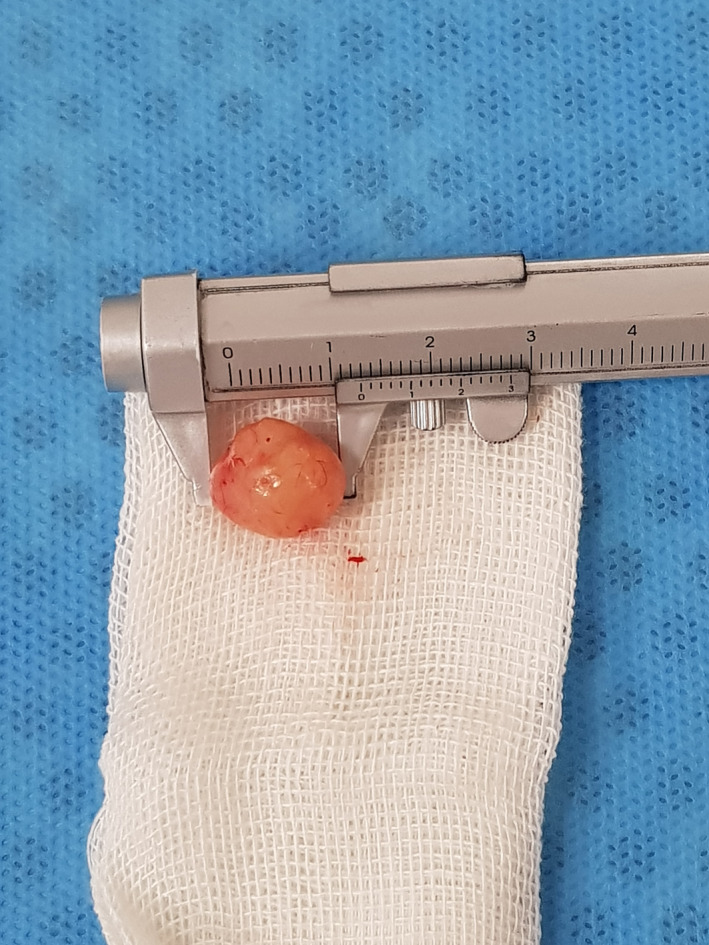
Biopsied specimen

**FIGURE 3:**
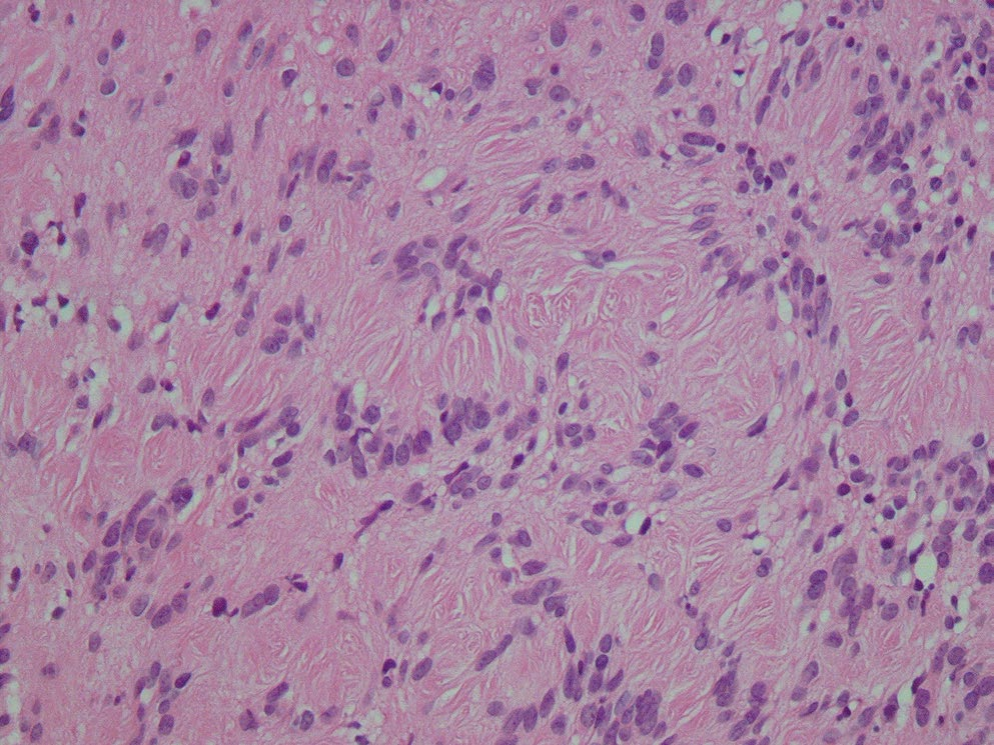
A benign neoplasm with a clear capsule composed of cells with spindle‐shaped nuclei, forming an Antoni A pattern

Based on the clinical findings (intact mucosal surface and no attachment to the epithelium) and the information acquired from the MRI, benign mesenchymal tumors were considered in the list of differential diagnosis. The fast growth rate of the tumor in our case was concerning. After obtaining written informed consent from the patient and his parents, the patient underwent excisional biopsy under local anesthesia by an oral and maxillofacial surgeon. The mass was submucosal, and after elevating a mucosal flap, a pedunculated yellow‐color mass with a smooth surface appeared, which measured 1 × 1 cm, and was easily resected with blunt dissection (Figure 2). The specimen was sent to the Pathology Department in formalin. The pathology report was as follows:

Microscopic examination revealed a benign neoplasm with a clear capsule composed of cells with spindle‐shaped nuclei, forming an Antoni A pattern. The tumoral cells did not show any cellular atypia or mitosis (Figure 3).

**FIGURE 4:**
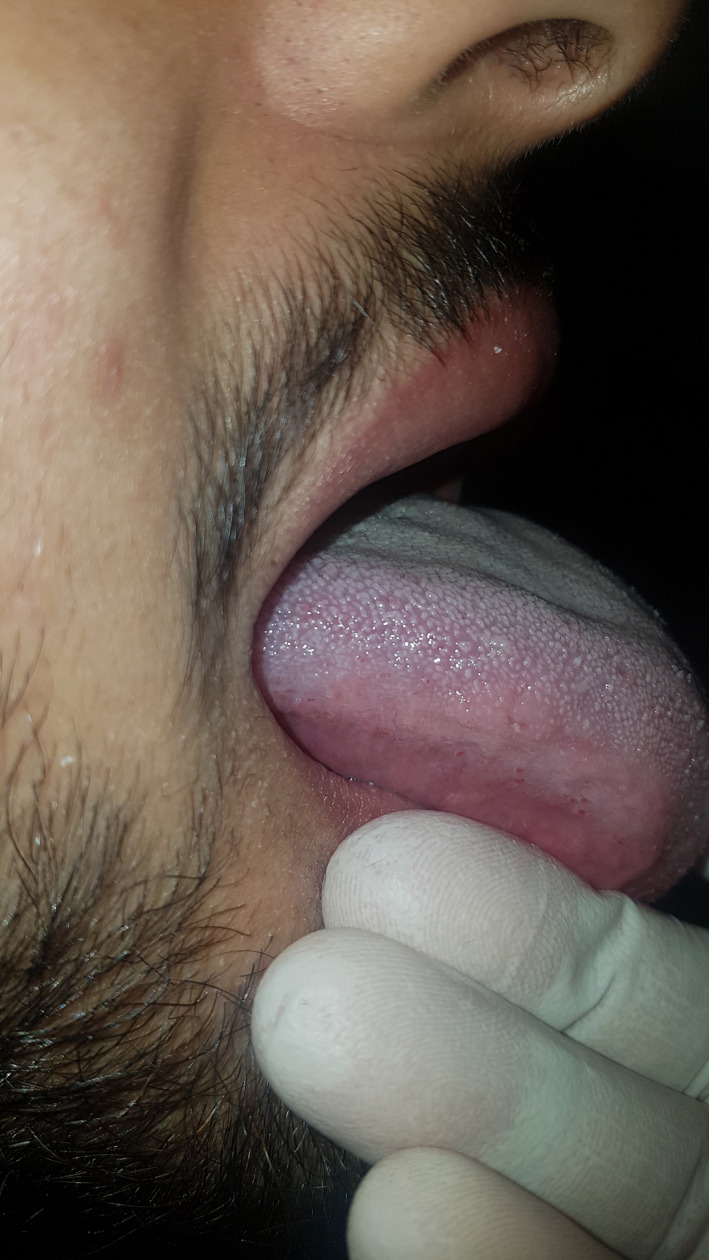
Postoperative view 1 year after surgery

Because of the benign nature of the lesion, no additional treatment was indicated. After the postoperative period, the patient's complaints, including the swelling, were resolved. The patient had no signs of recurrence in the first follow‐up session scheduled 1 year postoperatively (Figure 4).

## 3 DISCUSSION

Considering the electronic search for relevant articles published from 1954 to 2020, and by taking into account the current case, a total of 212 cases of oral schwannomas have been reported so far in the literature. The patients were classified into three groups of children, adolescents, and teenagers (≤20 years of age), middle‐aged individuals (>20 and ≤60 years), and the elderly (>60 years) based on their age range.

**FIGURE 5:**
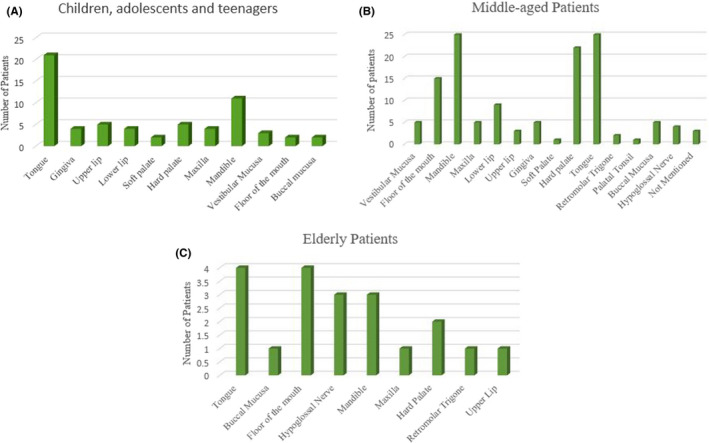
A, Location of lesions in children, adolescents, and teenagers. B, Location of lesions in middle‐aged patients. C, Location of lesions in the elderly patients

A total of 63 patients were included in the age‐group of children, adolescents, and teenagers (≤20 years of age); among which, 34 were males, 26 were females, and the gender of 3 had not been specified. Also, three patients in this group had ancient schwannoma. Figure 5A shows the location of lesions in patients in this age‐group.

A total of 129 patients were in the middle‐age group (>20 and ≤60 years); out of which, 65 were males and 64 were females. Figure 5B shows the location of lesions in middle‐aged patients.

A total of 20 patients were in the elderly group (>60 years); of which, 9 were males and 11 were females. Figure 5C shows the location of lesions in elderly patients.

Schwannoma or neurilemmoma is a benign tumor with slow growth, originating from the Schwann cells of the nerve sheath.^5^ Although 25%‐45% of all schwannomas occur in the head and neck region, intraoral schwannomas are rare, and only 1%‐12% of the head and neck schwannomas occur intraorally.^5^ The etiology of schwannomas is unknown. However, a number of factors such as spontaneous growth, extrinsic injury, chronic stimulation, and exposure to radiation have been suggested as possible etiologies.^6^

The chief complaint of patients in this age‐group was widely variable and the most common are included painless, tender, or painful swelling, which was fast‐growing or slow‐growing, dull pain in the jaw, an asymptomatic lesion detected during routine radiographic examination (intraosseous lesion), and hoarseness.

Microscopically, schwannomas are encapsulated tumors with two possible histological patterns of Antoni A and Antoni B. In the Antoni A pattern, the Schwann cells are spindle‐shaped and have elongated nuclei arranged in a palisading pattern around acellular areas (Verocay bodies). The Antoni B pattern has fewer cells with random orientation in a loose matrix.^7^

The current findings indicated that schwannoma may develop at a young age. Its prevalence was the highest in the middle‐aged individuals, and the lowest in the elderly. The tongue was the most common site of involvement in all age‐groups. The mandible was the second most common site of involvement in patients up to 60 years of age; while in the elderly, the floor of the mouth was the second most common site of involvement. Higher prevalence of schwannomas in the tongue may be due to the higher incidence of trauma to the tongue and the role of Schwann cells in repair of injured neurons.

Regarding the prevalence of schwannoma in different age‐groups, it seems that it has a slightly higher prevalence in males up to 60 years of age. However, this pattern changes after 60 years of age and it becomes slightly more prevalent in females.

Painless swelling with slow growth was the most common chief complaint of patients in all age‐groups.

Unlike other reports showing that the tongue is the most common site of involvement, it is not a common site of involvement for schwannoma in the Iranian population according to the existing literature. To the best of the authors' knowledge, the current case is the first case of tongue schwannoma in a 15‐year‐old Iranian male patient according to the existing literature (Table 1).

**TABLE 1:** Iranian patients with oral schwannomas reported in the literature

Author	Year	Gender	Age	Chief complaint	Location
Jahanshahi et al^8^	2011	Female	11	A swelling in the lower jaw with two‐month duration	Mandible
Rahpeyma et al^9^	2012	Female	12	A bulging in the soft palate developed three months earlier	Soft palate
Kargahi et al^10^	2012	Male	9	A swelling in the inferior border of the right mandible	Mandible
Khiavi et al^11^	2014	Male	21	Two‐month history of an asymptomatic mass in the palate	Hard palate
Yaghoobi and Pazyar^12^	2019	Male	16	A painless swelling of the lower lip	Lower lip
Present case	2020	Male	15	A painless swelling with one‐month duration	Tongue

Also, the chief complaint of our patient was a painless swelling with a fast growth, which was rare, compared with schwannomas reported in the literature. Since 1954, only six cases of oral schwannomas have been reported in the Iranian patients, including the current case.

## 4 CONCLUSION

In the Iranian population, Schwannoma is rarely included in the list of differential diagnosis of oral masses. Schwannoma can be considered as a differential diagnosis in a fast growth and painless swelling in an adolescent patient.

## CONFLICT OF INTEREST

None declared.

## AUTHOR CONTRIBUTIONS

All authors: contributed to the study conception. FA‐H, MY, and PA: performed data collections. MY and M‐SM: wrote the first draft of the manuscript. All authors: read and approved the final manuscript.

## ETHICAL APPROVAL

Written informed consent was obtained from the patient and his parents.

## Data Availability

The datasets supporting the conclusions of this article are included within the article and its additional files.

## References

[1] Harada H , Omura K , Maeda A . A massive pleomorphic adenoma of the submandibular salivary gland accompanied by neurilemomas of the neck misdiagnosed as a malignant tumor: report of case. J Oral Maxillofac Surg. 2001;59(8):931‐935. 11474457 10.1053/joms.2001.25035

[2] Ferner RE . Neurofibromatosis 1 and neurofibromatosis 2: a twenty first century perspective. Lancet Neurol. 2007;6(4):340‐351. 17362838 10.1016/S1474-4422(07)70075-3

[3] Armati P . The Biology of Schwann Cells: Development, Differentiation and Immunomodulation. Cambridge, UK: Cambridge University Press; 2007.

[4] Agha RA , Borrelli MR , Farwana R , et al. The SCARE 2018 statement: updating consensus surgical case report (SCARE) guidelines. Int J Surg. 2018;60:132‐136. 30342279 10.1016/j.ijsu.2018.10.028

[5] Cohen M , Wang MB . Schwannoma of the tongue: two case reports and review of the literature. Eur Arch Otorhinolaryngol. 2009;266(11):1823‐1829. 19130068 10.1007/s00405-008-0907-2 PMC2758150

[6] Hwang K , Kim SG , Ahn SI , Lee SI . Neurilemmoma of the tongue. J Craniofac Surg. 2005;16(5):859‐861. 16192870 10.1097/01.scs.0000164333.81428.f3

[7] Neville BW , Damm DD , Allen CM , Chi AC . Oral and Maxillofacial Pathology. Amsterdam, Netherlands: Elsevier Health Sciences. 2015.

[8] Jahanshahi G , Haghighat A , Azmoodeh F . Intraosseous neurilemmoma of the mandible: report of a rare ancient type. J Dent Res. 2011;8(3):150. PMC3177391 22013479

[9] Rahpeyma A , Jafarian AH , Ahmadi SK , Sarabadani J . A schwannoma of the soft palate in a child: histological and immunohistochemical features and surgical method. Iran J Otorhinolaryngol. 2012;24(67):95. 24303393 PMC3846209

[10] Kargahi N , Razavi SM , Hasheminia D , Keshani F , Safaei M , Hashemzadeh Z . Mandibular intraosseous schwannoma in a child: report of a rare case. J Dent Res. 2012;9(Suppl 1):S119. PMC3692190 23814552

[11] Khiavi MM , Zenouz AT , Mesgarzadeh AH , Sabetmehr O , Mahmoudi SM , Kouhsoltani M . Schwannoma in the midline of hard palate: a case report and review of literature. J Dent Res Dent Clin Dent Prospects. 2014;8(2):114. 25093057 10.5681/joddd.2014.021 PMC4120904

[12] Yaghoobi R , Pazyar N . Lower lip plexiform schwannoma: report of a rare case and a literature review. Indian J Dermatol. 2019;64(5):407. 31543538 10.4103/ijd.IJD_207_18 PMC6749764

